# Imperfect cross-linking of xanthan for pH-responsive bio-based composite moist wound dressings by stencil printing

**DOI:** 10.1177/08853282231210712

**Published:** 2023-11-06

**Authors:** Florian Wurm, Margit Lenninger, Astrid Mayr, Cornelia Lass-Floerl, Tung Pham, Thomas Bechtold

**Affiliations:** 1 Research Institute for Textile Chemistry and Textile Physics, 151267 University of Innsbruck, Dornbirn, Austria; University of Innsbruck, Innsbruck, Austria; 2 Hygiene and Medical Microbiology, 27280 Medical University of Innsbruck, Innsbruck, Austria

**Keywords:** Wound dressing, composite, bio-based, hydrogel, anti-microbial

## Abstract

The work addresses the use of bio-based and –degradable materials for the production of a moist, adaptive and anti-microbial wound dressing. The dressing is targeted to exhibit a pH-dependent active agent release. Xanthan hydrogel structures are coated on cellulose fabrics via stencil printing and subsequently cross-linked using glyoxal. By alteration of the cross-linker content from 1 to 6% by mass, the hydrogel elasticity can be tuned within a range of 2–16 kPa storage modulus. Increasing initial glyoxal concentrations also result in higher amounts of glyoxal release. Glyoxal, an anti-microbial agent with approval in veterinary medicine, is mostly released upon wound application supporting infection management. As wound simulation, normal saline, as pH 5 and pH 8 buffer solutions, were used. The release profile and magnitude of approx. 65%–90% glyoxal is pH-dependent. Increased release rates of glyoxal are present in pH 8 fluids, which mostly base on faster hydrogel swelling. Higher total glyoxal release is present in pH 5 fluid and normal saline after 3 days. Accordingly, a pH-dependent release profile was encountered. As glyoxal attacks any cell unselectively, it is expected to be effective against antibiotic resistant bacteria. By stencil printing the dressing size can be adjusted to minimize healthy glyoxal tissue exposure.

## Introduction

Healing and tissue growth depend on interconnected chemical, physical and physiological processes, which can be enabled and promoted by wound dressings. Primarily, wound dressings prevent further decontamination and physical wound contact. However, additionally they should neither adhere nor introduce toxic compounds, cause maceration and facilitate healing.^1^ Furthermore, many types of wounds also require moist healing conditions, e.g. burns, leg or pressure ulcers, as pressure application.^2^ Thus, composite structures consisting of a wound contact layer, keeping a moist milieu, supported by a secondary functional layer, preventing contamination and possibly enabling compression, are of interest. Additionally, wound dressings can be loaded with active substances which support antimicrobial wound management.^2,3^ Antimicrobial agents’ release profiles and doses are controlled via initial concentration, duration of dressing application as dressing’s constituents.

Chemically, wound infections and chronic wounds usually are accompanied with a more alkaline wound pH.^4–6^ It is therefore of interest to have a pH-sensitive release of microbial agents, with increasing release at increasing pH values. At normal pH the release of the antimicrobial agent is lowered, as to suppress prolonged application of the agents,^2^ or agent preservation.

Over the last decades biocompatibility and biodegradability become an issue.^7^ These properties can be accounted for by the use of biopolymeric dressings. Mono- and bilayer films from alginate and carboxymethyl cellulose (CMC) mixtures were found to produce flexible wound layers, absorbing large amount of fluids, while possibly serving as drug-release matrix.^7^ A similar approach was investigated by mixing alginate with gelatin.^3^ Usually, such biopolymer systems cover a secondary layer, which is supportive or moisture-blocking.^8^ The relatively low strength of the biopolymeric gels, can be adjusted using biopolymer mixtures,^3,7^ cross-linkers^9^ or increasing constituent’s concentration. Xanthan addition specifically has been investigated as a possible skin scaffold agent, due to its strength and biodegradability.^10^

With these arguments in mind we investigate an alternative composite wound dressing, consisting of a supportive cellulose fabric, with a moist xanthan hydrogel layer. The hydrogel is produced by the cross-linking xanthan using glyoxal, which is antimicrobial. Additionally, the release profile of cross-linked glyoxal is assumed pH dependant.

Cellulose fabric is chosen for biodegradability, biocompatibility and mechanical reasons.^11^ To sideglance, the fabric’s fluid uptake could be tuned by carboxymethylation if desired.^12^ Xanthan, due to its hydrophilic nature and tuneable attributes, has been investigate for several purposes, including oral application, tissue engineering and topical dressings.^13,14^ The similarity to cellulose, its widespread application,^15^ and the possibility to cross-link Xanthan using dialdehydes^16^ lead to the use of this biopolymer. Xanthan is cross-linked by glyoxal,^17^ to obtain a pH-dependent antimicrobial effect. Glyoxal is listed as biocide according to the European Chemical Agency,^18^ but is accepted in veterinary hygiene. Though glyoxal is present in human blood plasma at levels of 0.1–1 μmol/l, its also reported genotoxic, as tumour-promoting but not –initiating. It’s tolerable intake level is set to 0.2 mg/kg body weight per day for oral lifetime exposure.^19^ By glycation, it can also damage proteome and genome.^20^ Though these properties do not suggest human medical use, dialdehydes can be an option to tackle increasing antibiotic inefficacy, as upcoming bacterial silver resistivity. Besides, local and temporal glyoxal exposure can be adjusted to stay below persisting tissue effects.

Glyoxal molecules in the prepared composite are either un-cross-linked, serving as instant antimicrobial agent, are present as hemi-acetals, for possible retarded release by alkaline or acidic catalysis, or as acetals, for possible acidic catalysis. We hypothesize that acidic wound milieus result in increased overall glyoxal release, and that alkaline and acidic wound milieus generate different release profiles. With alkaline milieus favouring a faster release,^21^ which would be beneficial to counteract bacterial induced pH increases. Overall, we expect a pH-dependant behaviour of the glyoxal release.

Finally, the production of the dressings is performed via stencil printing. The printing area as the substrate are therefore easily adaptable. This enables minimizing the glyoxal-exposed tissues areas, as adapt wound dressing size for veterinary application.^22^

The study therefore focuses on the cellulose-xanthan composite wound dressings, cross-linked using glyoxal. Investigate were the swelling behaviour of the dressings, the glyoxal release, as its antimicrobial efficacy. Glyoxal release was investigated using normal saline, or wound pH buffer solutions of pH 5 and pH 8.

## Materials and methods

### Materials

Food-grade xanthan from Jungbunzlauer Suisse AG (Basel, Switzerland), 30% glyoxal-solution, di-potassium hydrogen phosphate, potassium dihydrogen phosphate from Merck KGaA (Darmstadt, Germany), magnesium chloride hexahydrate, hydrochloric acid, sodium acetate, acetic acid from Carl Roth GmbH + Co. KG (Karlsruhe, Germany), sodium chloride from Zeller GmbH (Hohenems, Austria) and sodium hydroxide from Deuring GmbH & Co. KG (Hörbranz, Austria) were used as received. Deionized (di) water was delivered from a stationary ion exchanger (conductivity < 5 μS/cm). Liquid ammonia treated cotton fabric St 41 from Veramtex S.A. (Brussels, Belgium) was used for FT-IR, HPLC and swelling determinations. Antimicrobial samples were prepared from a cotton Achim fabric (Getzner Textil AG, Bludenz, Austria).

### Sample preparation

Xanthan gum solutions were blended with respective amounts of glyoxal and di water to obtain a final 3% wt. xanthan concentration of desired glyoxal (0%–12% by mass) concentration. Into this solution magnesium chloride was added (additionally 1%), and formulations were left stirring for 3 min. The obtained pastes were used for stencil printing (polytetrafluoroethylene stencil of 5 × 5 × 0.1 cm). A second set of pastes was printed using a bigger stencil (7.5 × 7.5 × 0.2 cm) on clear film for subsequent swelling and rheometric gel characterisation.

All printed samples were oven-dried at (45 ± 3)°C overnight. Cross-linking was performed in a Mathis Lab. Dryer (Oberhasli, Switzerland) at 140°C for 3 min. We did not include samples with glyoxal but without the final cross-linking step, as we expect even faster release profiles. Such profiles were not the goal of the approach, as a latent and retarded release was of primary interest. Afterwards, samples were kept in polypropylene bags until further characterisation.

### FTIR, optical microscopy, SEM

Fourier transform infrared (FTIR) spectroscopy characterisation of the samples were recorded from the imprinted side of the fabrics, using an attenuated total reflection (ATR) unit on a Bruker Invenio FTIR spectrometer (Billerica, USA). Presented data is the mean signal of three samples. Scanning electron micrographs were recorded using a TM 4000 from Hitachi Ltd. Corporation (Tokyo, Japan) applying a 10 or 15 kV electron acceleration voltage.

### Swelling

Dried samples were cut to 4 × 4 cm, and immersed in normal saline (0.9% sodium chloride (NaCl)), pH 5 or pH 8-buffered fluid. Normal saline was chosen as simple mimic of physiological fluids. Swelling was investigated using 3 samples of each glyoxal concentration. pH 5 fluid was prepared using a 0.1 mol/l acetate buffer, pH 8 by a 0.1 mol/l phosphate buffer. The samples were taken out of the swelling fluid at distinct swelling times for weight determination. Excess fluid was softly stripped and dripped off, to not damage the samples.

### Rheometry

Prepared samples were immersed in normal saline for 1 h before their characterisation using serrated parallel plates (50 mm diameter) on a Anton Paar (Graz, Austria) MCR 302 rheometer. Dynamic moduli were determined from 2 min (10 Hz, 0.3% shear) measurements, interrupted by an intermediate frequency sweep (0.3%, 0.016–30 Hz). Gap height was set manually and data acquisition was initiated once normal force dropped below 1 N. An error of 5% on the different moduli is imposed, a value taken from experience. Visco-elastic properties were evaluated only once, as pastes were homogenous and sample preparation straightforward.

A dilution series of xanthan solutions in 0.1 mol/l NaCl in deionized (di) water was used to estimate the molecular weight of the polymer using a parallel plate system (25 mm diameter). Solutions were introduced in the rheometer using a pipette, three equivalent runs per sample were conducted, and obtained intrinsic viscosity estimates were drawn from the mean value of a Huggins and Kramer plot evaluation to zero concentration. Finally, applying the Mark-Houwink equation led to estimated molecular weight.^23^ Xanthan molecular weight of the samples was estimated to (2140 ± 530) kg/mol.

### HPLC

Glyoxal concentration was determined from the swelling solutions of samples. In a first run, samples were immersed in 40 mL solution for up to 30 h. Regularly, 6 mL solution were drawn for solid phase extraction (SPE) on a Macherey- Nagel (Düren, Germany) Chromabond SB column and subsequent high pressure liquid chromatography (HPLC) determination. The columns were conditioned with 5 mL of pH 8 phosphate buffer, and components were retrieved using 5 mL of 5 mmol/l sulphuric acid. A second glyoxal elution determination was performed after 74.5 h, without subsequent SPE, in 20 mL solution. After drying of these samples they were reimmersed for >30 min in 15 mL di water, before addition of 5 mL 4 mol/l hydrochloric acid and heating to 85°C for 20 min. A final glyoxal determination was undertaken.

HPLC analysis was performed using an Aminex 87H column in a Shimadzu Nexera XR (Kyoto, Japan) instrument. Sample constituents were separated at 65°C, a flow of 0.8 mL/min and in injection volume of 20 μL, using the method proposed by Zhang et al.^24^

### Antimicrobial efficacy

*Staphylococcus aureus* (*S. aureus*, ATCC 29,213), *Escherichia coli* (*E. coli*, ATCC 25,922) and *Pseudomonas aeruginosa* (*P. aeruginosa*, ATCC 27,853) grown on Columbia agar with 5% sheep blood (Becton-Dickinson, Franklin Lakes, NJ, USA) at 35 ± 2°C were selected as test organisms because they are known to be responsible for burn and wound infections.^25,26^ Each inoculum was prepared by transferring a 24-h-old colony into 2 mL of distilled water and adjusting it to a McFarland standard turbidity equivalent of 0.5 (≈1.5 × 10^8^ colony forming units cfu/mL) using DensiCHEK Plus (bioMérieux, Marcy l’Etoile, France).

The agar diffusion test (Kirby-Bauer) test procedure was carried out as previously described.^27,28^ Briefly, Mueller- Hinton agar plates (Becton, Dickinson and Company, Franlin Lakes, USA) were inoculated with the corresponding bacterial suspension in three directions to form a confluent lawn. Then, dressings ((2.5 ± 0.2) cm × (2.5± 0.2) cm squares), containing the compound at various concentrations (1%, 6% glyoxal) were placed on the agar surface. Cellulose squares without any active compound served as negative control. After incubating the plates for 24 h at 35 ± 2°C, the inhibition zone diameters were evaluated. Each test consisted of at least three replicates per dressing type and mean values were reported. The results were expressed in millimetres and mean average was calculated. A larger zone diameter correlates to higher antimicrobial activity.

## Results

**Figure 1.**
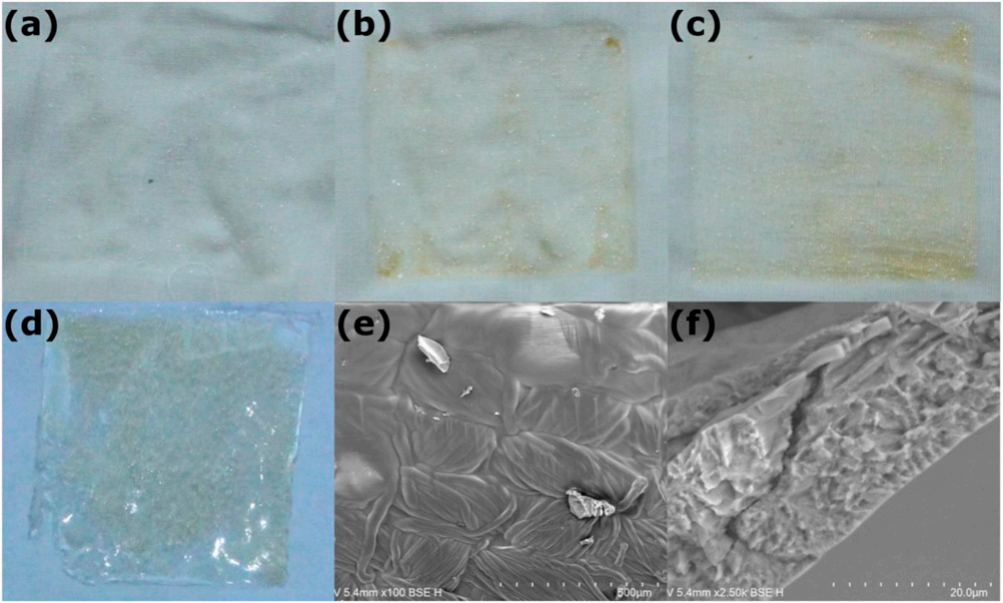
Upper row: Xanthan-cellulose fabric samples containing (a) 0%, (b) 1% and (c) 6% glyoxal by mass. Lower row: (d) Xanthan structures with 6% by mass glyoxal peeled from clear foil for rheometry (e) SEM image of 6% by mass glyoxal sample surface and (f) a cross-section of a dried xanthan layer containing no glyoxal.

The samples show different shades of coloration (Figure 1). The inhomogeneous shading derives from the heated air flow during cross-linking inside the lab dryer as fluid migration effects in drying, which drives polymer degradation non-homogenously. We do not expect this to deteriorate the dressing’s effectiveness.

The mass of the prepared samples can be compared with the theoretical mass of the samples (Table 1). We compare sample mass at laboratory conditions (24 ± 2°C, 50 ± 10% RH).

**Table 1.** Weight comparison of the samples’ theoretical dry masses with the measured dry masses of a 4 cm × 4 cm piece.

Sample (% mass glyoxal)	Theoretical (g)	Measured (g)	Deviation (%)
Cellulose fabric	0.192	0.200 ± 0.005	4.5 ± 0.1
0	0.282	0.278 ± 0.028	−1.6 ± 10.1
0.5	0.291	0.293 ± 0.034	0.8 ± 11.7
1	0.299	0.281 ± 0.024	−6.0 ± 8.7
6	0.385	0.391 ± 0.035	1.7 ± 8.9

### Infrared analysis

**Figure 2.**
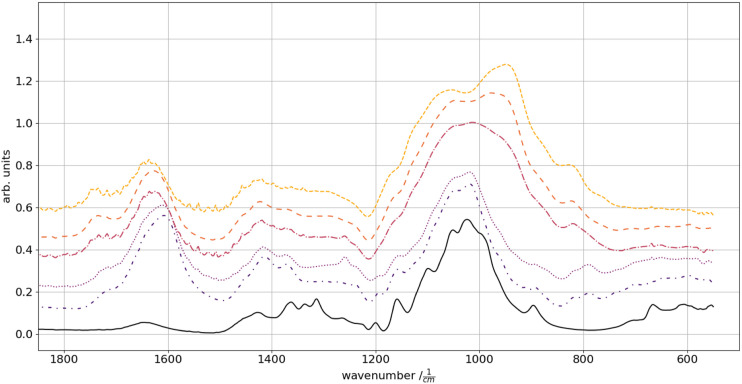
FTIR spectra of a pure cellulose fabric (solid) as prepared samples of increasing glyoxal concentration from bottom to top: 0% (dash-dot-dotted), 1% (dotted), 2% (dash-dotted), 6% (loosely dashed) and 12% wt. (dashed).

Infrared analysis was undertaken to verify and determine the presence of xanthan-ester formation, as the presence of the di-aldehydes. The signals reveal a relative change in the band for asymmetric COO^−^ vibration (∼1600 cm^−1^), a minor contribution to the symmetric COO^−^ (1430 cm^−1^) and a pronounced superposition of the C-O stretching vibration (1025 cm^−1^) by the stretching of the C-O-C (945 cm^−1^)^29,30^ with increasing glyoxal content (Figure 2). This is based on glyoxal oligomer formation in drying solutions.^19,31^ The unobtrusive change in the 1740 cm^−1^ peak derives from C=O stabilization changes. Since this peak is present in the aldehyde as the xanthan ester, the shift towards higher wave numbers can be contributed to the shift of the C=O towards unbound glyoxal compared to the ‘stabilized’ xanthan ester with a lower wave number. A minor increase in the absorption dedicated to acetals, due to the superposition of the C-O stretching in the region of 1060–1160 cm^−1^, is present.^32^ Here it can be an indicative for xanthan cross-linking.^33^ The asymmetric COO^−^ (1590 cm^−1^) vibrations become less prominent with increasing glyoxal content. In parallel, they shift from ∼1590 to ∼1635 cm^−1^. Accordingly, the ketonic CH_2_ symmetric deformation (1410 cm^−1^) perishes with glyoxal increases.

There are no prominent differences in the FTIR signals between samples cross-linked for 3 min each at 120°C, 130°C or 140°C. Therefore, 140°C was chosen as cross-linking temperature.

### Swelling

**Figure 3.**
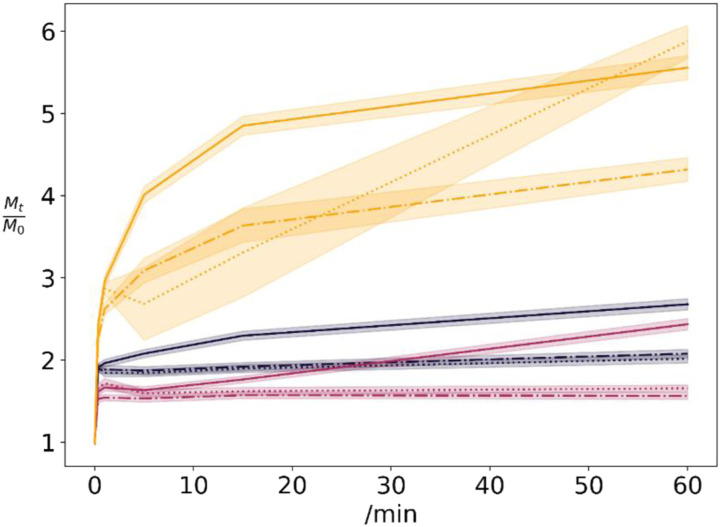
Normalized swelling sample weights of 0% (yellow), 1% (grey) and 6% (red) by mass glyoxal samples for swelling in normal saline (dot-dashed), pH 5 (dotted) and pH 8 (solid) media. Shaded areas represent standard deviations of 3 repetitions.

Swelling assays were conducted, to estimate the water holding capacity of the hydrogel structures and their respective moist state in a dressing (Figure 3). Samples without glyoxal exhibits the largest extent of swelling. The collapsed and dried xanthan structures reforms a hydrogel matrix. That in turn induces the printed layer to grow and increase in height. No swelling differences for the structure were optically perceivable.

For many samples, an initial weight loss is perceived upon immersion in swelling fluid. This weight loss is present in the minor dip of the higher glyoxal-concentration curves. Presumably, this initial weight loss is by glyoxal, magnesium chloride and xanthan wash-off.

### Rheometry

**Figure 4.**
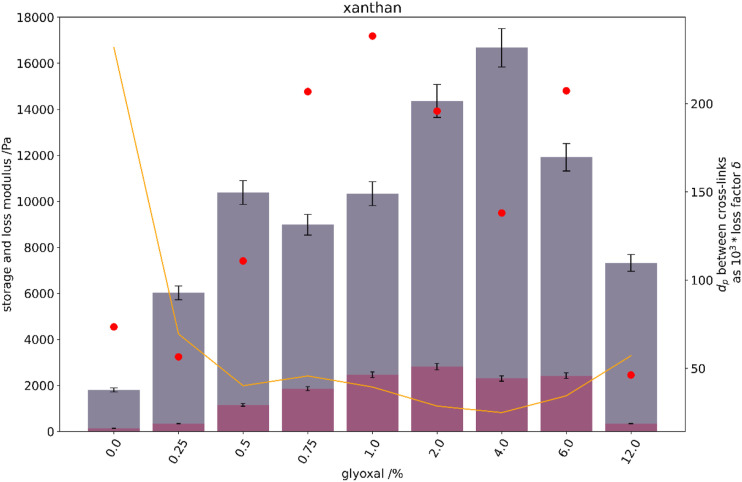
(Left axis) bars of storage (petrol) and loss modulus (red) of xanthan hydrogel structures at 10 Hz, 0.3% shear, given a 5% error. (Right axis) theoretical degree of polymerisation (D_p_) between cross-links (yellow solid line) as a multiple of the loss factor: 1000*δ (red dots).

To evaluate the hydrogel elastic and viscous properties, the partially swollen xanthan gels were evaluated by the use of a rheometer. Visco-elastic properties were evaluated after 1 h of swelling in normal saline. With increasing cross-linker in the xanthan structures, the elasticity, represented by the storage modulus, increases (Figure 4). It reaches a maximum for a glyoxal content of 4% by mass, after which elasticity decreases. Cross-linking based on entanglement and hornification of the glyoxal-free xanthan structures, already results in relatively low elastic xanthan gel structures. These neither dissolve in normal saline, nor break under mild compression or while handling.

Using the theory of elasticity,^34^ the degree of polymerisation between cross-links was estimated (equation (1)):

(1) $$G_{s}=\frac{G^{\prime }}{\cos\;\delta }\text{and}\;N_{mm}=\frac{c*R*T}{G_{s}*M_{w}}$$

G_s_ is the shear modulus, G′ the storage modulus obtained from analysis, δ the loss factor, c the concentration of the polymer, R the gas constant, T the temperature, M_w_ the monomer weight and finally N_mm_ the estimated number of monomers between cross-links. For the monomeric weight, it is assumed that half of the anhydroglucose units have an acetyl and pyruvate group attached.

### HPLC

The quantify the glyoxal wash-off, and thereby the present anti-microbial agent concentration, the swelling media was evaluated by high-pressure liquid chromatography, following the method of Zhang et al.^24^ Most of the glyoxal is lost in the SPE (Table 2).

**Table 2.** Determined hplc limits of detection for direct injection and SPE-extracted calibration solution.

System	Direct injection	SPE	Zhang et al 2013^24^
LOD (mg/l)	31.99	166.77	0.06

**Figure 5.**
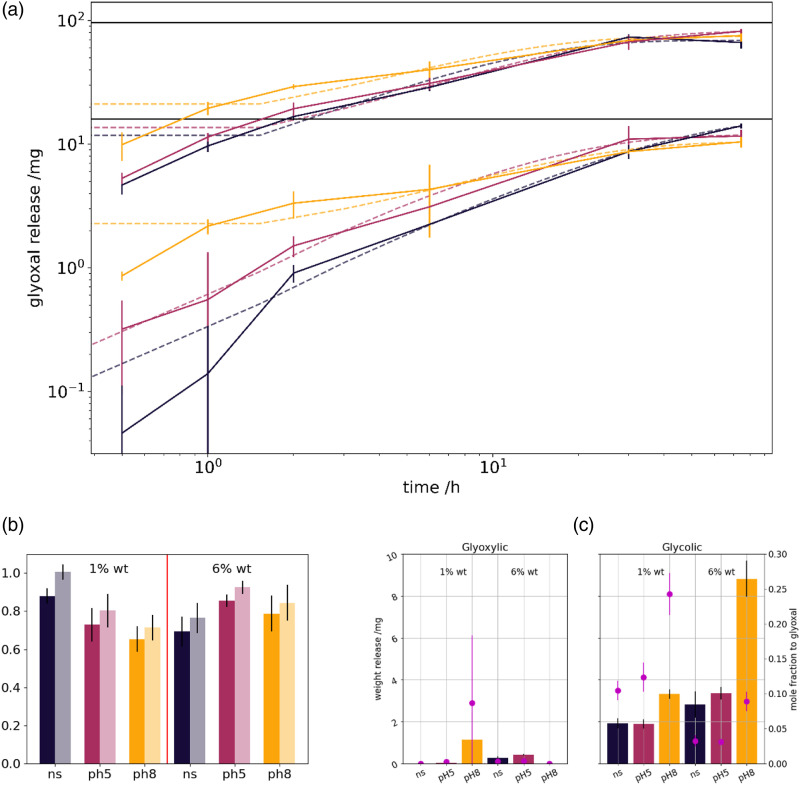
Elution media for all subplots: (grey) normal saline, (red) pH 5 and (yellow) pH 8-buffered media. (a) Glyoxal release of 1% (lower curves) and 6% (upper curves) by mass glyoxal dressings. (b) Share of total glyoxal released (left bar – strong color), as with an additional 1 mol/l HCl wash (right bar – light shade) (c) glyoxylic acid mass release (left) and glycolic acid mass release (right) with given mol ratio to total glyoxal (violet dots).

The elution profile from 1% to 6% glyoxal samples in the different swelling media is presented in Figure 5(a).

The amount of glyoxal detected in the swelling solutions can be compared with the absolute amount of glyoxal present in the dressings (Figure 5(b)). Alongside minor amounts of glyoxylic and glycolic acid are detected, which most likely form upon glyoxal oxidation, as in the de-cross-linking reaction (Figure 5(c)). Obviously, in pH8 media, more glyoxylic as glycolic acid is being formed. Their share compared to the glyoxal release increases in the hydrochloric acid wash (data not shown).

### Antimicrobial property

Obviously, the following test are to prove the dressings’ anti-microbial efficacies. Distinct antimicrobial activity was identified in the case of dressings containing 1% and 6% glyoxal against all tested microorganisms, while no antimicrobial activity was noticed for negative control (cellulose dressings).

**Figure 6.**
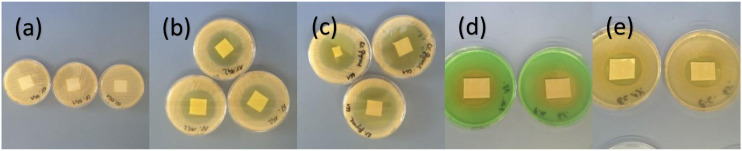
Agar diffusion tests after 24 h incubation: (a) 0% glyoxal, (b) 1% glyoxal or (c) 6% glyoxal against *S. aureus*, (d) 1% (left) and 6% (right) glyoxal against *P. aeruginosa*, and (e) 1% (left) and 6% (right) glyoxal against *E. coli*.

The diameters of the inhibition zones were against *S. aureus*, 42 ± 3.6 (1%) and 52.6 ± 3.1 (6%), against *P. aeruginosa*, 41.6 ± 2.5 (1%) and 43.6 ± 3.2 (6%), and against *E. coli*, 38.6 ± 1.5 (1%) and 39 ± 3.6 (6%) mm, respectively. However, it should be noted, that compared to *E. coli* and *P. aeruginosa* the effect on *S. aureus* was slightly higher (Figure 6).

From the elution profile one can estimate that a 2.5 cm × 2.5 cm dressing pieces elute (2.8 ± 0.4) mg and (25.2 ± 3.8) mg for a 1% and 6% by weight glyoxal dressing in normal saline.

## Discussion

Sample coloration derives from the acid degradation of the xanthan and cellulose polymers^35,36^ and chromophore formation in the presence of glyoxal.^37^ During preparation before the cross-linking step at 140°C and after drying over-night the samples are uncoloured. This colour vanishes with solution immersion. Samples with catalyst but without glyoxal do not show any or very little shading, which indicates that glyoxal in combination with an acid catalyst is a prerequisite for coloration. Eventually, leaching of degradation products and catalyst results in initial mass loss of the samples in swelling,^38^ which goes optically undetected due to fluid dilution.

Minor sample mass deviations, due to humidity, from the theoretical mass values exist. We did compare sample mass after crosslinking with values after adaption to lab. conditions for 24 h and found only minor weight changes in the region of ±0.003 g, independent of glyoxal concentration. In lab. atmosphere the cellulose fabric has a humidity content of (4.5 ± 0.1) % by mass (oven dried for 4 h at 110°C). We desisted from exact sample moisture content determination by extensive drying to prevent additional hornification. As mass values match we assume that little to no glyoxal is lost during the preparation procedure.

For estimation calculation yields that in 1% by mass glyoxal gels the ratio of glyoxal to xanthan anhydroglucose unit is (1.0 ± 0.1), while it is (5.5 ± 0.5) in 6% by mass glyoxal samples. This derives under the assumption that half of the xanthan side chain contain both an acetyl and a pyruvate group.

### Swelling

Swelling properties of the samples differ comprehensively. Due to hornification induced by drying, which includes esterification of xanthan’s acid and alcoholic groups,^39^ even pure xanthan gels do not dissolve. Interestingly, all samples having more than 1% wt. glyoxal, do reach an intermediate swelling equilibrium within the first hour, supposedly within the first 15 minutes. For samples of lower glyoxal concentration, the swelling is more prominent within the first hour.

In pH 8 buffer, the glyoxal-free sample seems to swell constantly, probably as hornfication-induced hydrogens bonds break up. These samples swell faster than in pH 5 media. This is probably due to increased electrostatic repulsion.^39^ In pH 5 media the samples do equilibrate rapidly, except for the 0% glyoxal dressing. We assume that acetal and hemiacetal cross-links are only slowly being hydrolysed.

After the first hour subsequent swelling is present. In normal saline and pH 5-buffer, this weight gain is (41 ± 14) % over more than 20 h of swelling. It seems that glyoxal-free samples, and 12% wt. glyoxal samples, are above this value, but we do not have enough data to substantiate that. In pH 8 buffer, swelling is decelerating over these 20 h to weight gains of (14 ± 15) %. Accordingly, weight gains for 6% wt. glyoxal samples were (29 ± 29) %, compared to (14 ± 3) % for glyoxal-free samples and (3 ± 5) % for 1% wt. glyoxal samples. We interpret, that the few cross-links in lower concentrated samples are rapidly de-cross-linked, while the higher glyoxal samples require more time until cross-links release.

Based on the equivalent buffer strength, we do not expect major differences in the hydrodynamic properties of the xanthan chains.^23^ Additionally, pH 5 and pH 8 are well above the pK_a_ of the present carboxylic and pyruvate groups. We accordingly assume most of these groups to be deprotonated. Samples with the addition of 10% by mass magnesium chloride catalyst were also produced, as this was reported to inhibit hornification.^40^ However, xanthan structures readily dissolved in swelling media.

We summarise the swelling to be pH-dependant, with faster swelling in higher pH media. However, the pH dependant swelling decelerates while lower pH samples keep swelling.

### Rheometry

Xathan gels behave primarily elastic. At low glyoxal concentration a slight increase in cross-linker has a distinct effect on hydrogel elasticity with increasing storage modulus. The theoretical number of the degree of polymerisation between cross-links also decreases, as more links are formed and these are shorter distanced.

Samples with increasing glyoxal content were very stiff and hard to handle as they were foil like and easily broke. It is therefore counter-intuitive, that the storage modulus of these samples should decrease. Most probably, these gels had relaxation time constants substantially higher than the measuring frequency. The serration of the rheometer plates indented the structures, and these did only partly relax until the subsequent oscillation re-stressed the gel. Accordingly, the storage modulus of the samples is recorded to be lower than it actually is. Possibly, the oscillation frequency should have been reduced to 1 Hz, or even below. This was neglected as the handling of these films was difficult. Besides, in our understanding these samples are of little importance for the use case.

For comparison reasons, all samples were rheologically characterized after 1 h swelling in normal saline, though this biases the less elastic samples to more elasticity. After the first hour, the majority of the swelling has appeared. We therefore assume that this swelling state is a possible state for comparison. As gel structures soften with time, the rheometric comparison of final swelling equilibria is difficult due to several gels becoming unmanageable.

Swelling of the samples correlates with the findings of the visco-elastic determination (see Figure 4). Swelling of a polymer network is the response of the solvation of polymers and ions, the entropy of polymer bundling and the shear relaxation process. Equilibrium is achieved once shear modulus prevents the additional inflow of the swelling agent. With an increase in cross-links, the magnitude of water absorption usually depletes.^41^

Xanthan is usually used for thickening purposes and has been cross-linked by several methods. In the absence of a specific investigation, we can compare our values with the cation-effects on 1% xanthan gels. These range from 0.03 to 13 kPa in the presence of Fe^3+^ and Na^+^.^42^ In the absence of iron or calcium, 3% xanthan concentration and given glyoxal cross-linking, hydrogel storage moduli of several kPa seem reasonable compared to effective Fe^3+^ cross-linking.

Summarising, hydrogel structures are present for all samples. As the lower glyoxal samples result in relatively soft samples, which easily break, a minimum glyoxal content of 1% by mass was chosen as a lower working limit.

### Hplc

Elution profiles of the swelling samples enable several conclusion. There is little difference between the glyoxal concentration in normal saline and pH 5-buffered solutions. Initially, in pH 8 solutions higher amounts of glyoxal are eluted. Accelerated swelling enables un-cross-linked glyoxal to solubilize faster. Furthermore, it bases on the higher reaction rate constant of the alkaline de-cross-linking^43^ of hemi-acetals. Acetal release requires a two-step process. In acidic conditions after the first hydrolysis of the acetal to the hemi-acetal, the usually faster hemi-acetal hydrolysis can proceed. There is two necessary reactions to release acetal glyoxal. However, acidic conditions enable acetal release, and finally more glyoxal is released.^43^

Obviously, elution differences are lower for 1% by mass glyoxal dressings, and increases for 6% by mass glyoxal dressings. We expect the pH 5 samples to release more glyoxal compared to the normal saline ones, however this is not found in 1% by mass samples.

Cross-linked glyoxal is expected to form esters with xanthan and can accordingly be compared to cross-linked citric acid with cellulose. Schramm et al found approx. 71%–77% butanetetracarboxylic acid and 53%–68% citric acid bound on cellulose fabrics, recuperable by alkaline hydrolysis. The non-bound acids were washed-off priorly.^44^ Glyoxal release shares of up to 90%, as mentioned above, are therefore comparable, as the un-bound glyoxal is additionally present.

The difference in our limit of detection (LOD) compared to Zhang et al. probably derives from the use of an aged HPLC column.^24^ Since measured concentrations were several orders of magnitude above our LOD, we desisted from optimizing it. We further assume the hemi-acetals to from on C2 and C3 of the related glucose, mannose and glucuronic residues, as vicinal hydroxyl groups are more likely to hemi-acetylise.^17^

Recollecting, we recorded a media-dependent varying elution pattern, with initial increased release rates at increase pH. This corroborates the pH-dependant release hypothesis. Thus, the dressing can act as a ph-responsive release agent.

### Antimicrobial testing

To evaluate the in vitro antimicrobial activity of the dressings disk diffusion testing, a common, inexpensive and simple method to determine antibacterial effects, was performed.^45,46^ However, this qualitative test registers only antimicrobial materials that contain a diffusible compound and does not yield quantitative assessment. Additional complicating factors imply the visual reading and partially indistinct inhibition zones leading to poor reproducibility of the results.^47^ Antibacterial activity was estimated by applying one single method, though different method-material combinations might influence method outcome.^47^ Potential influences of relevant factors that could affect microbiocidal activity, i.e. relative humidity or temperature were not evaluated in this study. The applicability of the in vitro findings with regard to antiseptic efficacy and influence on wound healing needs to be further investigated by combining in vitro results with future clinical studies.

One can compare the size of the inhibition zones of the antimicrobial test with known reported values from the applied protocol.^27^ *Stapphylococcus* specia are classified susceptible to 30 μg Cefazolin with inhibitions zones ≥18 mm, *Pseudomonas aeroguinosas* to 30 μg Cefotaxime with zones ≥23 mm, and *E. coli* to 10 μg Ampicillin with zones ≥17 mm. According to these values, above reported 1% and 6% glyoxal wound dressings inhibition zones imply anti-microbial efficacy comparable with selected antibiotic species.

We have not studied antibiotic resistant bacterial strains. These are part of a follow-up study. However, we project that several bacterial antibiotic counter-measures are inefficient against glyoxal.^48^ Due to the high reactivity and the small size of the aldehyde, efflux pumps, specific inactivating enzymes, cell wall protein modifications or modified drug targets are expected inefficient, or an overdo. Glyoxal is estimated to unselectively attack several cell constituents, including the cell walls, without selectively interrupting specific cell processes. It thereby weakens present cells via multiple pathways, rendering selective counter-measures rather ineffective. However, the cell attack is unselective and also affects healthy and wanted cells.

## Conclusion

Hydrophilic xanthan structures were cross-linked onto cellulosic fabrics, using stencil printing. Glyoxal works as xanthan cross-linker and antimicrobial agent. The respective xanthan’s gel strength, as the glyoxal release, can be adjusted, but partly correlate. It is therefore reasonable to assume that for the sake of antimicrobial management, the glyoxal release profile is adjusted, while the gel strength follows.

Most notable, the glyoxal release profile from the wound dressing is pH-dependent, and thereby responsive. An attribute which is beneficial, as wound pH increases are reported in several bacterial wound infections.^4–6^ The initial excess glyoxal release at higher pHs can serve as a desirable on-demand medication. In normal saline and pH 5 wound milieu constant glyoxal release enables antimicrobial management. This can for example be of interest in wounds where subsequent contamination is non-negligible. This could be the case in veterinary setting, where wound cleaning as hair removal might be difficult, or animals nibble on dressings. Likewise in human medicine, where patients with itching wounds cannot desist to scratch and stress. The current release profile seems to cover 1–3 days, depending on the required glyoxal dose.

Disadvantageously, glyoxal release correlates with increasing xanthan gel softness, and can accordingly lead to its mechanical disintegration. It also limits its applicability in pressured applications. Possibly, xanthan concentration increases, or use of citric acid as secondary cross-linker,^49^ can counter-act this. This in return changes swelling properties and release profiles, as dressing effectiveness, and has to be anticipated. Negatively noted also, that glyoxal due to its ambivalent properties is not accepted for human applications yet.

Several limitations were introduced during this study, of which few are mentioned. Methodically, glyoxal oxidation could have been used to obtain higher retention shares in the SPE procedure. Additionally, more wound exudate fluid-like swelling solutions could have been applied. However, we do not expect the glyoxal release to alter extensively by these. The adhesion strength of the xanthan gel structure on the cellulose matrix would be an interesting characterization to determine the composites integrity. Lap shear and scrapping test might be tools to characterize this property.

Anticipating, di-aldehydes crosslink wound exudate amino acids, as amino acid containing compounds, and possibly accelerate the formation of a wound covering layer.^50^ Furthermore, in human and in the veterinary setting of interest, the form and size of the wound dressing can be altered by the printing stencil. The dressings can be sterilised and dry-stored, decreasing transport weight and increasing storage time. Finally, glyoxal could be an alternate approach for infection management if microbial antibiotic and silver resistance are further progressing.
